# Visible Light-Activated Persulfates Towards Dyes Decolorization: Performance, Kinetics, and Statistical Optimization

**DOI:** 10.3390/ijms27156620

**Published:** 2026-07-24

**Authors:** Piotr Zawadzki, Łukasz Pierzchała

**Affiliations:** Central Mining Institute—National Research Institute, Plac Gwarków 1, 40-166 Katowice, Poland; lpierzchala@gig.eu

**Keywords:** methylene blue, advanced oxidation processes, visible light-activated persulfates, response surface methodology, optimization

## Abstract

This article aims to investigate the effects of operational factors on methylene blue (MB) decolorization in a visible light advanced oxidation process (AOP). Response surface methodology was applied to evaluate variable interactions and identify the best operational conditions of the process. The response surface analysis of the expected increase in methylene blue decolorization at different levels of the variable parameters was employed. The experiment evaluated the influence of the following parameters: pH, glucose dose, Na_2_S_2_O_8_ (SPS) concentration, and initial dye concentration. The model confirmed that the optimal conditions for MB decolorization in the visible light-driven advanced oxidation process were: SPS dose = 30 mM, glucose dose = 230 mM, pH = 4. The highest decolorization level was obtained at a concentration of C_0[MB]_ = 5 ppm (R = 63%). The kinetics of the process followed pseudo-first-order (R^2^ = 97–99%). The radical scavenger test showed that both sulfate and hydroxy radicals are involved in MB decolorization. The test confirmed that the proposed method is effective even at a broad range of MB concentrations (1–10 ppm). The analysis showed that the proposed method is highly efficient across various dye concentrations, which is particularly significant from a practical perspective. Summarizing, this study fills a research gap in the optimization of methylene blue decolorization within the scope of advanced oxidation processes driven by visible light and glucose, demonstrating high efficiency across a range of different environmental variables. The study showed that the SPS/Vis/glucose process can serve as a valuable alternative to conventional decolorization methods.

## 1. Introduction

Methylene blue (MB) is broadly applied in the textile, paper, cosmetics, plastics, and food industries. MB is a cationic phenothiazine dye containing a six-membered heterocyclic ring molecule with sulfur and a nitrogen atom (phenothiazine ring) [1]. Due to its extensive industrial use, MB may occur in dye-containing wastewater discharged from manufacturing, dyeing, washing, and rinsing operations. Even at low concentrations, MB can impart intense coloration to water, reduce light penetration, and disturb photosynthetic activity in aquatic environments [2]. Methylene blue has been reported to exert toxic effects on aquatic organisms, while its biologically active structure requires careful consideration when assessing environmental and health-related risks [3].

To meet the constantly increasing requirements for treated wastewater and potable water quality, it is necessary to apply state-of-the-art processes. Hence, advanced oxidation processes (AOPs) play a key role in this regard [4]. AOPs are highly significant in providing water that is safe for human health and free from both microbiological hazards and micropollutants, such as dyes. The common feature of AOPs is the chemical reaction between organic pollutants and hydroxyl radical (•OH) or sulfate radical (${SO}_{4}^{•-}$) [5]. •OH and ${SO}_{4}^{•-}$ radicals differ from other oxidants mainly due to their high oxidation potential (E^0^). For example, the oxidation potential of ozone, one of the strongest oxidants used in water and wastewater treatment, is E^0^ = 2.08 V in an acidic medium. Meanwhile, the oxidation potential of a hydroxyl radical is E^0^ = 1.8–2.7 V, while for a sulfate radical it is E^0^ = 2.5–3.1 V [6].

Sulfate radicals have become particularly significant for dye decolorization in AOPs [7,8,9]. To generate ${SO}_{4}^{•-}$ radicals, its precursor (e.g., persulfate—PS) needs to be activated. Without activation, persulfate can react only with some organic compounds, and the process efficiency is much lower compared to activated persulfates. Without activation, the persulfate anion $\left(S_{2}O_{8}^{2-}\right)$ exhibits an oxidation potential lower by about 33% compared to the sulfate radical ${SO}_{4}^{•-}$ [10]. Compared to the •OH radical, the ${SO}_{4}^{•-}$ radical reactivity is independent of pH. The •OH radical reactivity decreases together with the increase in pH (e.g., the optimal pH for the Fenton reaction is about 3) [11]. At a neutral pH, the ${SO}_{4}^{•-}$ radical is more reactive than •OH [12]. This is a significant advantage because it eliminates the need to apply additional chemical agents to adjust the wastewater pH. This can be problematic, especially in cases involving major streams or wastewater that is significantly buffered.

A treatment process conducted in the presence of PS is an efficient and effective method for pollutant degradation, but it requires expensive chemical reagents or energy-consuming UV lamps. The disadvantage of activation by UV radiation is the power of the used lamps, which often exceeds 100 W and may sometimes reach even up to 500 W [13]. Alkaline activation (pH > 11) requires significant amounts of chemical reagents to achieve and maintain the high pH and, simultaneously, pH regulation to a value required as per applicable regulations. Transition metal ion activation involves costs, not only related to activator reagents but also to pH correction. For example, the price of FeSO_4_·7H_2_O, a compound commonly used for PS activation, can be twice as high as that of simple sugars (such as glucose). The disadvantage of using transition metal ions (e.g., ferrous ions Fe^0^, Fe^2+^) is the generation of iron sediments, which necessitates the additional separation of the resulting suspensions and their further disposal. Eliminating the metal ions after the process can also be expensive, as toxic metal ions, such as cobalt ions, are also used for activation purposes [14].

A technically simple and metal-free activation strategy involves persulfate activation through the combined use of an organic promoter (glucose) and readily available visible light (Vis). Organic promoters primarily reduce operating costs and facilitate sodium persulfate (SPS) activation by visible light. Combining processes of glucose and visible light with AOPs shortens the process time and increases contaminant decolorization efficiency. Glucose is an optically active substance; it is an electron donor for SPS since the carbon material activation mechanism is possible. It is worth highlighting that glucose is a biodegradable, plant-based organic activator. The EC_50_ for glucose in aquatic plants (algae) and the LC_50_ for arthropods (daphnia) are 1.9 × 10^6^ mg/L and 4.03 × 10^6^ mg/L, respectively. Electricity cost plays a key role in the economic viability of water and wastewater treatment, as energy consumption is a primary factor in municipal facility operating costs. Therefore, prioritizing solar-driven AOPs research is essential, as visible light provides a free, renewable energy source.

Numerous authors have conducted studies on the optimization of methylene blue decolorization using response surface methodology (RSM). For example, ref. [15] investigated the effects of flow rate, initial concentration, pH, titanium(IV) oxide dosage, and UV radiation on methylene blue decolorization without a catalyst. The methylene blue decolorization was performed in a photocatalytic reactor using TiO_2_ as a catalyst and fluorinated ethylene propylene tubing as the UV radiation transmitter. The test demonstrated that reactor efficiency also depended on the flow rate (optimal at 10 mL/min) and solution pH (optimal at pH = 7). The analyzed variables included initial dye concentration, TiO_2_ dose, pH, and solution flow rate. On the other hand, ref. [16] presented a methodology for methylene blue decolorization using a Vortex Fluidic Device coupled with UV light via the photo-contact electrification mechanism. The analyzed variables included rotational speed, time, and dye concentration. In the study [17], the authors synthesized a graphitic carbon nitride photocatalyst for the photocatalytic decolorization of MB. The synergistic effect of photocatalyst dose, pH, and irradiation time was statistically studied and optimized.

A significant knowledge gap is the lack of systematic optimization frameworks to optimize the interactions between critical variables that affect the activation of persulfate-AOPs by a non-toxic, biodegradable plant-based organic promoter (glucose) and visible light. This gap limits the ability to evaluate the potential and limitations of glucose-assisted visible-light persulfate activation under controlled model solution conditions. This research addresses the identified gap by implementing a model-based calibration framework. A primary distinguishing aspect of this work is the use of response surface methodology (RSM) to investigate and optimize the relationships between input variables and responses, thereby determining the expected model equations. The effects of pH, glucose dose, Na_2_S_2_O_8_ concentration, and initial dye concentration are comprehensively evaluated, offering new insights into the mechanisms of glucose-assisted visible light persulfate activation.

Industrial dyeing wastewater exhibits considerable pH variability, typically ranging from pH = 4 to 9. Most AOP studies are limited to near-neutral or alkaline conditions, leaving a critical gap in understanding process performance under real industrial conditions. The optimization of AOPs in acidic conditions, which are essential for fiber dyeing, has been largely overlooked. This study presents an advancement in visible light-driven AOPs through systematic process optimization across a wide pH range (pH from 4 to 9), with particular emphasis on acidic conditions (pH = 4) representative of wool, alpaca, and mohair dyeing. For example, dyeing wool requires an acidic environment, as high pH can damage the fibers [18,19]. The structural composition of methylene blue (a cationic dye) allows it to react with the acid group present in the fibers, generally forming electrovalent bonds [20]. Acid-treated fibers are characterized by durability and intense colors.

Therefore, this study was undertaken to address the lack of systematic optimization strategies for visible light-assisted persulfate activation using biodegradable organic promoters. The work focused on the development and assessment of a SPS/Vis/glucose process for methylene blue decolorization through response surface methodology (RSM). The effects of pH, glucose dose, SPS concentration, and initial dye concentration were investigated, with particular emphasis on acidic conditions relevant to real industrial fiber dyeing processes. Furthermore, kinetic analyses and radical scavenger experiments were performed to evaluate reaction pathways and identify the dominant oxidizing species. The findings provide new insights into glucose-assisted persulfate activation and support the application of the SPS/Vis/glucose system as an alternative treatment option for dye-containing wastewaters.

## 2. Results

### 2.1. Influence of SPS Concentration

**Figure 1:**
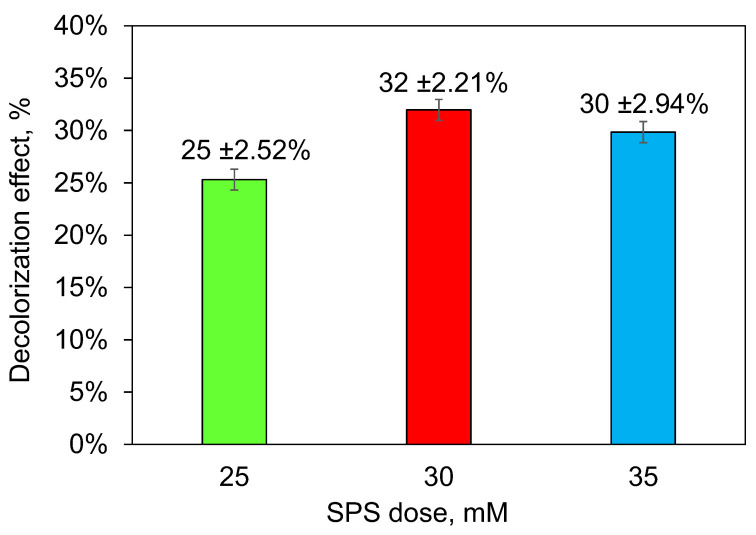
Decolorization effects under the influence of different SPS concentrations. Experimental conditions: pH = 6, glucose concentration = 230 mM, C_0[MB]_ = 5 ppm.

The SPS dose has a significant effect on MB decolorization. Figure 1 shows that after 70 min, the decolorization efficiency reached a level of 25–32%. The highest decolorization level was obtained at an SPS dose of 30 mM. A higher SPS dose results in lower decolorization efficiency due to a reaction between the sulfate anion and hydroxyl radicals. This phenomenon is characteristic of MB decolorization processes involving both persulfates and photocatalytic methods, and it also occurs with other dyes (e.g., acid orange) [21]. It is primarily related to potential particle aggregation or sulfate radical oxidation by other oxidizing forms, such as S_2_O_8_^2−^. Ref. [22] demonstrated that increasing the catalyst dose can reduce light penetration in a dye solution in the heterogeneous photocatalytic oxidation process. Similar findings were reported by [23,24].

### 2.2. Influence of Glucose Concentration

**Figure 2:**
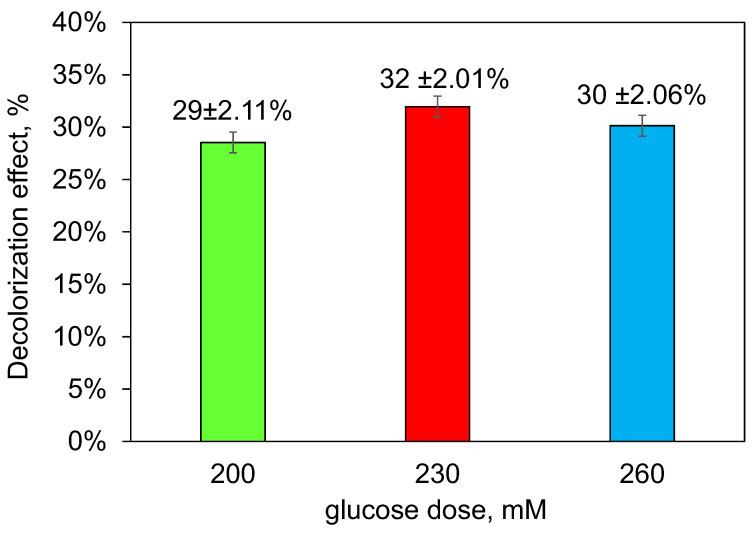
Decolorization effects under the influence of different glucose concentrations. Experimental conditions: pH = 6, SPS concentration = 30 mM, C_0[MB]_ = 5 ppm.

Glucose was applied to activate the SPS. Figure 2 shows that after 70 min, the decolorization efficiency reached a level of 29–32%. The highest decolorization level was obtained at a glucose dose of 230 mM. Exceeding the optimal dose leads to a reduced decolorization level. The inhibition of the decolorization effect results from the properties of glucose, which also serves as a free radical scavenger kOH•=1.5·109 M/s [25]. Glucose, like other sugars (e.g., sucrose), is used to activate persulfate in visible light through the mechanism of carbon material activation [26]. Glucose is an electron donor, and the activation mechanism is similar to that of phenoxides, as described in the publication [27]. The electron from glucose is transferred to the persulfate, thereby activating it, whereas the glucose is oxidized into products that may activate the persulfate.

### 2.3. Influence of MB Concentration

**Figure 3:**
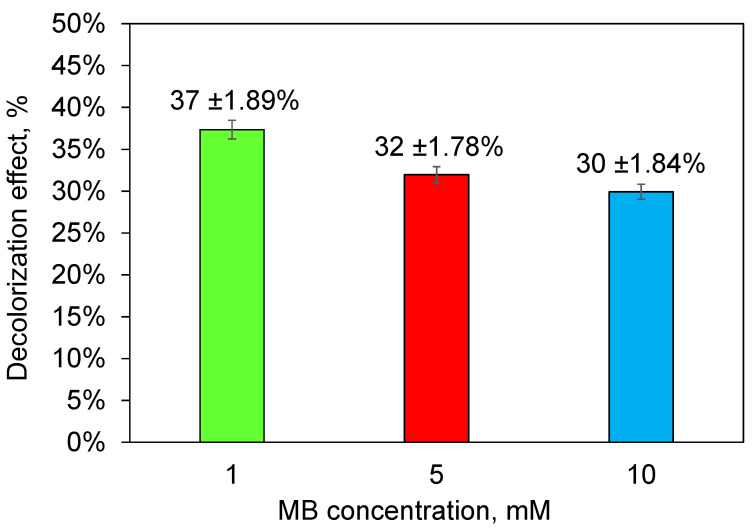
Decolorization effects under the influence of different MB concentrations. Experimental conditions: pH = 6, SPS concentration = 30 mM, glucose concentration = 230 mM.

An increase in MB concentration disrupts processes involving oxidizing radicals. Figure 3 shows that after 70 min, the decolorization efficiency reached a level of 30–37%. The highest decolorization level was obtained at a concentration of C_0[MB]_ = 1 ppm. The decolorization level decreases as the concentration increases. The phenomenon can be observed in cases of other AOPs, as well as those involving persulfates [28]. The disruption of decolorization by increasing dye molecule concentration can be attributed to several factors. The use of oxidizing radicals increases with the increase in concentration. Therefore, the likelihood of a collision between the oxidizing radicals and the dye molecules decreases [29]. In light-driven AOPs, the most likely reason for this is that light cannot penetrate solutions at high concentrations, which ultimately leads to lower oxidizing radical production [30]. Furthermore, intermediate compounds can consume oxidizing radicals, which results in poor decolorization (competition effect). The experiment demonstrated that the SPS/Vis/glucose process exhibits similar efficiency over a broad range of MB concentrations, from 1 to 10 ppm.

### 2.4. Influence of pH

**Figure 4:**
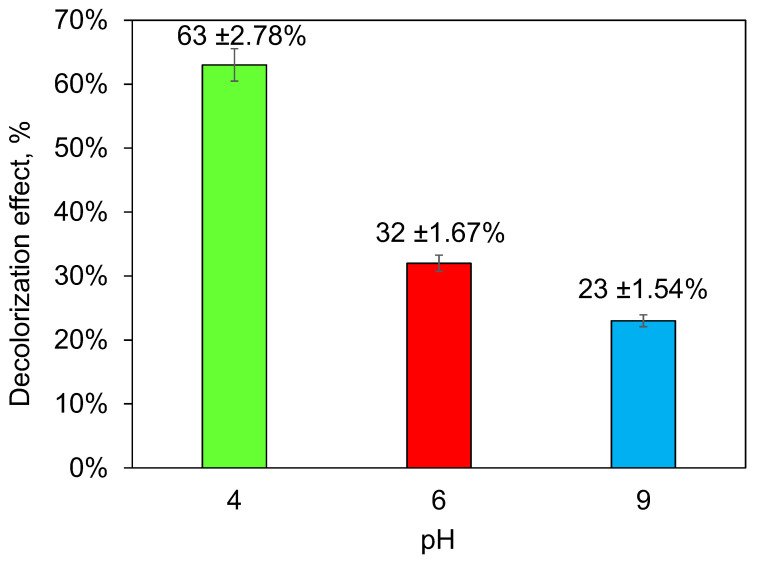
Decolorization effects under the influence of different pH levels. Experimental conditions: SPS concentration = 30 mM, glucose concentration = 230 mM, C_0[MB]_ = 5 ppm.

The solution pH is one of the key parameters that affect the oxidation of organic pollutants. The following pH values were tested: 4, 6, and 9. Figure 4 shows that after 70 min, the decolorization efficiency ranged from 23% to 63%. The highest decolorization level was achieved at pH = 4. pH is a similarly significant parameter in processes involving SPS, as well as in photodegradation processes using photocatalysts (e.g., TiO_2_). In both cases, the pH affects the contribution of individual oxidizing radical forms. For SPS, the ${SO}_{4}^{•-}$ radical reactivity is generally independent of pH [31], but various oxidizing forms predominate depending on the pH. Although the oxidation potential changes together with pH, persulfate-based systems exhibit a broad range of pH applications. For example, the study by [32] confirmed that reaction efficiency decreased with an increase in pH.

### 2.5. Radical Scavenger Tests

**Figure 5:**
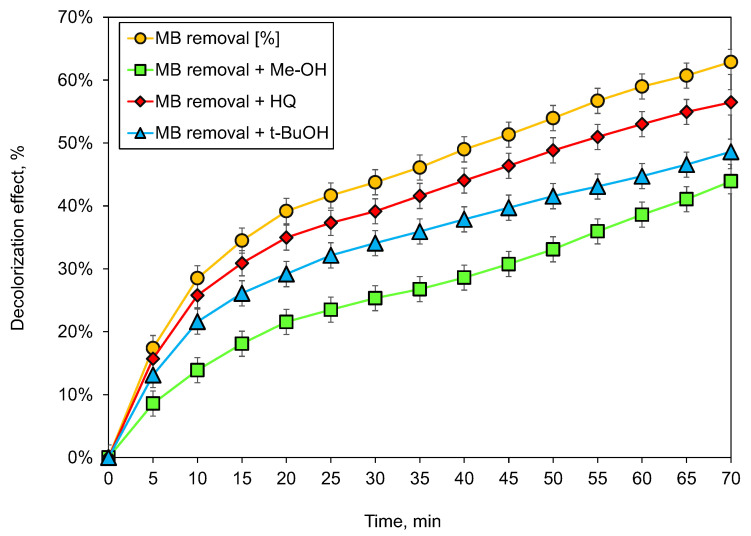
Decolorization effects under the influence of different radical scavengers. Experimental conditions: SPS concentration = 30 mM, glucose concentration = 230 mM, pH = 4, C_0[MB]_ = 5 ppm.

The radical scavenger test showed that both ${SO}_{4}^{•-}$ and •OH radicals are involved in methylene blue decolorization (Figure 5). The decolorization was scavenged primarily by the ${SO}_{4}^{•-}$ radical scavenger (Me-OH), while •OH radicals also participated in the decolorization of MB. $O_{2}^{•-}$ radicals participated in the reaction to the most minor extent. Methanol (Me-OH) is a scavenger of •OH (kOH•=8·108 M/s) and ${SO}_{4}^{•-}$ ($k_{{SO}_{4}^{•-}}=1\cdot 10^{7}\;M/s$). Tert-butyl alcohol (t-BuOH) is a scavenger of •OH radicals (kOH•=5.2·108 M/s). The decolorization of MB was scavenged mainly due to the Me-OH (approx. 53% in the first 15 min), while due to t-BuOH, it was approx. 29% in the first 15 min.

The use of AOPs based on •OH radicals is generally limited due to the need to ensure acidic conditions (pH = 2 to 4) and the instability of H_2_O_2_. As shown in the study [33], in an acidic environment, the activity of PS and PMS is favorable. In less favorable alkaline conditions, ${SO}_{4}^{•-}$ radicals are converted into •OH radicals as a result of reaction with $O_{2}^{•-}$ radicals (Equations (1) and (2)). These results indicated that the decolorization of methylene blue occurred in the presence of strong oxidizing radicals like ${SO}_{4}^{•-}$, generated by visible light activation of SPS.

(1) $$O_{3}^{-}S-O-O-{SO}_{3}^{-}+H_{2}O\overset{{OH}^{-}}{\to }2{SO}_{4}^{2-}+{HO}_{2}^{-}+H^{+}$$

(2) $$O_{3}^{-}S-O-O-{SO}_{3}^{-}+{HO}_{2}^{-}\to {SO}_{4}^{2-}+{SO}_{4}^{•-}+O_{2}^{•-}{+H}^{+}$$

As shown above, ${SO}_{4}^{•-}$ radicals dominate at pH = 4, while $O_{2}^{•-}$ and •OH occur in alkaline conditions. The reaction of $O_{2}^{•-}$ with ${SO}_{4}^{•-}$ radicals generates •OH radicals, with the proportion of $O_{2}^{•-}$ radicals being the smallest.

The persulfate hydrolysis produces the HO_2_^−^ anion, which then reacts with the persulfate molecule, resulting in the ${SO}_{4}^{•-}$ and $O_{2}^{•-}$ radicals. The increase in the pH of the reaction medium results in the reaction of the ${SO}_{4}^{•-}$ with H_2_O or OH^−^ and the production of hydroxyl radicals (Equations (3) and (4)).

(3) SO4•−+H2O→HSO4−+OH•

(4) SO4•−+•OH−→SO42−+OH•

### 2.6. Kinetics Studies

**Figure 6:**
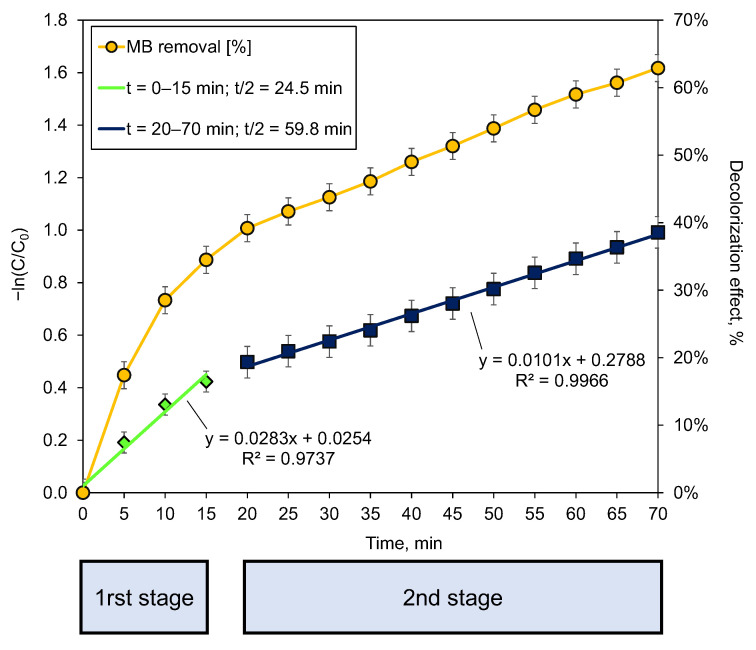
Kinetic constants. Experimental conditions: SPS concentration = 30 mM, glucose concentration = 230 mM, pH = 4, C_0[MB]_ = 5 ppm.

The kinetic model is divided into two stages (Figure 6), which are related to indirect oxidation reactions of pollutants. The methylene blue decolorization curve in the first stage (t = 0–15 min) and in the second stage (t = 20–70 min) fitted well with the pseudo-first-order model, showing a very good fit of experimental data (both stages R^2^ = 97%). This confirms the validity of using pseudo-first-order reaction kinetics as a model suitable for the removal of low-concentration pollutants [34,35,36]. For example, in the publication [37], a two-stage model of phenolic compound degradation in the persulfate activated with manganese oxide ordered mesoporous carbon composites was adopted. This was related to the accumulation of phenol molecules on the catalyst surface in the first stage of the reaction, followed by phenol exhaustion and reaction slowdown. In turn, in the publication [38], authors demonstrated that caffeine degradation also proceeded in two stages in accordance with pseudo-first-order reaction kinetics.

The reaction kinetics confirm that the MB decolorization intensity is highest in the first 15 min. The mathematically calculated half-life was determined to be t/2 = 24.5 min. The pseudo-first-order reaction kinetics confirms that ${SO}_{4}^{•-}$ radicals are primarily involved in the decolorization (Table 1), as previously confirmed. The ${SO}_{4}^{•-}$ radical scavenger (Me-OH) scavenges the first phase of the process (up to 15 min) by approx. 53% and by approx. 35% during the second phase (up to 70 min). In contrast, $O_{2}^{•-}$ radicals were scavenged by HQ the least. HQ slowed decolorization by 13% and 18% after 15 min and 70 min, respectively. This explains the benefits of decolorization at pH = 4 since ${SO}_{4}^{•-}$ radicals dominate, whereas the •OH radical generation is lower due to the lowest $O_{2}^{•-}$ radical participation. Acidic pH promotes the stability of ${SO}_{4}^{•-}$ radicals and minimizes the weakening conversion of ${SO}_{4}^{•-}$ to •OH.

### 2.7. Optimization of Methylene Blue Decolorization

**Figure 7:**
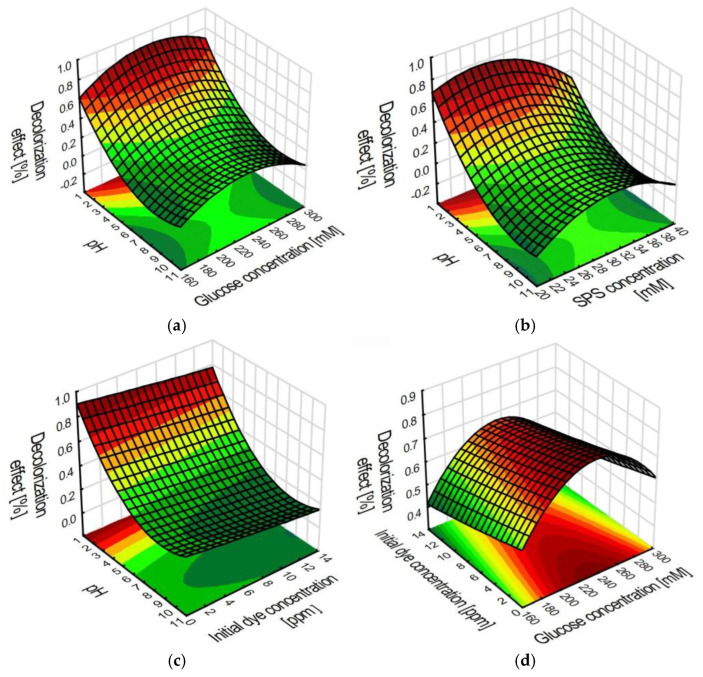

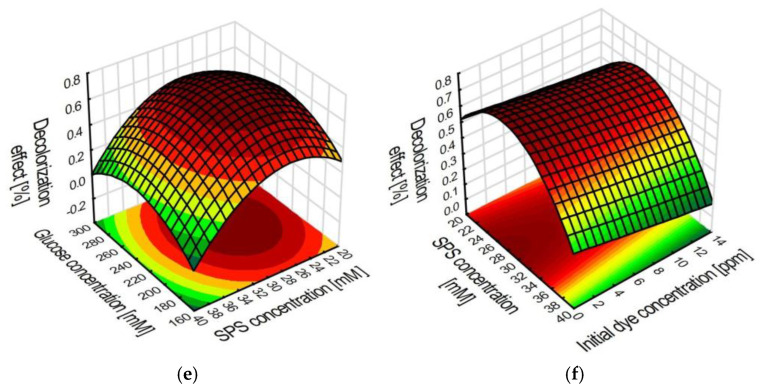
Response surface plots for decolorization effect (%) and interaction factors: (**a**)—pH and glucose concentration, (**b**)—pH and SPS concentration, (**c**)—pH and MB concentration, (**d**)—MB and glucose concentration, (**e**)—glucose and SPS concentration, (**f**)—SPS and MB concentration.

Figure 7 illustrates the effects of process variables (pH, glucose dose, SPS dose, and initial dye concentration) on the effectiveness of the MB decolorization method, which involves a visible light-driven advanced oxidation process. Figure 7a,b present 3D plots of the MB decolorization effect (%), respectively, for the glucose dose and pH, and the SPS dose and pH. The results demonstrate that the decolorization effect increases with decreasing pH and is optimal at a glucose dose of 230 mM, as confirmed by decolorization efficiency analyses. The decolorization effect decreases with increasing pH due to the ${SO}_{4}^{•-}$ radical being trapped by •OH radicals, which are dominant at higher pH levels [39]. Another reason is the state of the methylene blue charge (cationic form at pH levels of 1.7–8) [40] and the dissociation of SPS into S_2_O_8_^2−^ ions with two negative charges, which results in electron transfer from the dye to SPS, which is in turn responsible for the decolorization effect. It is also affected by the selected persulfate-based oxidation system, as well as the type of pollutants being removed. The test also confirmed that the proposed method is effective at a broad range of MB concentrations (1–10 ppm). The analysis demonstrated that the proposed method at low pH levels is highly efficient across various dye concentrations, which is particularly significant from a practical perspective, as shown in Figure 7c. Generally, the proposed method was equally efficient at both low (1 ppm) and high dye concentrations. The model analysis also reveals that an optimal glucose dose was selected for MB concentration. Glucose enables persulfate activation and the generation of oxidizing radicals, as documented in publications [41,42]. Figure 7d illustrates that an optimal MB reduction was achieved at a glucose concentration of 230 mM, consistent with the sodium persulfate dose (Figure 7f). Figure 7e shows that the efficiency of the method increases at central values of SPS and glucose dose. The optimal determined doses were 30 mM and 230 mM for SPS and glucose, respectively. The model confirmed that the optimal conditions for MB decolorization in the visible light-driven advanced oxidation process were as follows: SPS dose = 30 mM, glucose dose = 230 mM, and pH = 4. The highest decolorization level was achieved at a concentration of C_0[MB]_ = 5 ppm; however, the SPS/Vis/glucose process exhibits similar efficiency over a broad range of MB concentrations, from 1 to 10 ppm. This demonstrates the significant advantages of the tested process, as it is resistant to variations in MB concentration and capable of efficient solution decolorization. For example, 0.63 ppm of the dye was removed at C_0[MB]_ = 1 ppm, while a similar efficiency was obtained at a concentration of C_0[MB]_ = 10 ppm, with as much as 5.2 ppm of the dye removed. Textile industry wastewater can be characterized by low pH [43] and variable pH depending on the applied processes [44].

## 3. Discussion

The study demonstrated that the SPS/Vis/glucose process can be a valuable alternative to conventional decolorization methods (Table 2). Existing methods demonstrate an MB decolorization efficacy of R = 28–72% obtained under various laboratory conditions, which complicates a direct comparison of the results. Various MB decolorization technologies (e.g., ozonation, UV/H_2_O_2_, Photo-Fenton, photocatalysis) were compared under different environmental conditions. However, existing studies do not reveal a clear prevalence of experiments conducted at acidic or alkaline pH levels. For example, at pH = 4, an efficiency of 61% was achieved during ozonation [45] whereas at pH = 11, the efficiency was 71% during photocatalysis [46]. Regarding the reported methods, the SPS/Vis/glucose process is a promising method for MB decolorization over a wide pH range. Table 2 demonstrates the advantage of the SPS/Vis/glucose process over UV/H_2_O_2_. The UV/H_2_O_2_ process exhibits lower efficiency (50%) compared to SPS/Vis/glucose at both pH = 4 [47] and pH = 11 [48]. SPS/Vis/glucose process avoids the formation of iron oxide sediments compared to [49], which uses the Fenton reagent. The primary disadvantages of the Fenton reagent are the relatively high costs of H_2_O_2_ and the generation of iron oxide sediments. SPS/Vis/glucose is also strongly competitive with photocatalytic processes [50]. Its main advantage is the lower energy consumption thanks to the use of low-power lamps (10 W). This study highlights the practical significance of the pH of industrial dyeing wastewater, as it has real environmental conditions (pH approx. 4). Therefore, the added value of this research is not only high effectiveness over a wide pH range, but above all, the high performance at acidic pH, which reduces the need to dose external reagents that affect treatment costs.

## 4. Materials and Methods

### 4.1. Materials

The aqueous solutions were prepared by dissolving a methylene blue (MB) standard at concentrations of 1–10 ppm (purity > 97%) in deionized water. Such a dye concentration can be identified in wastewater from the textile industry. It was assumed that the decolorization of wastewater containing methylene blue at a concentration of 1–10 ppm is to be applied for the tertiary treatment of wastewater containing low concentrations of MB (average of about 5 ppm), whose treatment in conventional systems yields poor effects. The model solution pH (pH = 4–9) was corrected using 0.1 mol/L HCl or 0.1 mol/L NaOH. The MB decolorization was performed using sulfate radicals. The source of the oxidizing radicals was sodium persulfate (SPS). The SPS activation was done using visible light (Vis) and glucose (≥99.5%). All the chemical reagents used during the experiment were obtained from Merck Life Science (Poznań, Poland).

### 4.2. Experimental Procedure

**Figure 8:**
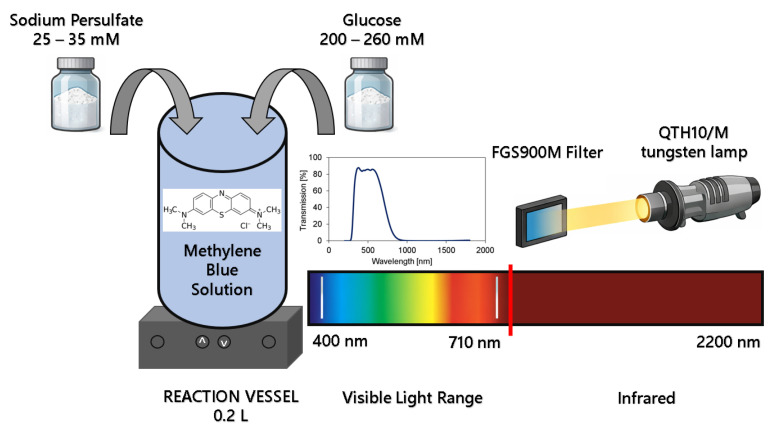
Experimental setup.

The UV-Vis absorption spectra of methylene blue were studied within a range of 200–700 nm using a V-750 spectrophotometer (Jasco, Cracow, Poland). The solutions were measured at the wavelength λ_max_ = 665 nm. Solutions with absorbance higher than 1 were appropriately diluted to enhance the sensitivity of quantitative analysis. The source of the sulfate radicals was sodium persulfate (SPS). The SPS dose was 25–35 mM. The SPS activation was performed using visible light (Vis) with a wavelength of >400 nm and glucose. The glucose dose was 200–260 mM. The experiment setup is shown in Figure 8. The source of visible light was a QTH10/M tungsten lamp with a power of 10 W (Thorlabs Inc., Newton, NJ, USA). The lamp emitted light with a wavelength λ = 400–2200 nm. An FGS900M filter (Thorlabs Inc., Newton, NJ, USA) was used to cut off the light spectrum bands over 710 nm. All experiments were performed in vessels with a volume of 0.2 L. The process was carried out in darkness to eliminate additional light sources. All the advanced oxidation processes were conducted for 70 min. A magnetic stirrer was used to ensure optimal mixing of the model solution. The temperature (T = 298 K), reaction (pH = 4–9), atmospheric pressure (P = 1013 hPa), and reaction vessel volume and shape (cylindrical borosilicate glass beakers) were the same during each experiment. All experiments were performed in triplicate. The mean values ± standard deviation are shown in the figures.

As part of the preliminary studies, basic control experiments were conducted, and the effects of various methylene blue (MB) decolorization configurations were examined: MB alone, visible light alone, SPS alone, glucose alone, SPS + visible light, SPS + glucose in the dark, glucose + visible light without SPS, and SPS/Vis/glucose. The results are summarized in Supplementary Materials (Table S1).

The objective of this study was to evaluate the loss of visible color caused by methylene blue chromophore transformation. The use of UV-Vis absorbance is appropriate for monitoring MB decolorization because MB exhibits strong absorption in the visible range and the decrease in absorbance directly reflects the loss of color intensity.

The degree of MB decolorization was determined based on characteristic absorption peaks at λ_max_ = 665 nm. In order to determine the decolorization efficiency (*D_E_*), the absorption was measured before and after AOP, as shown in Equation (5), where A_0_—initial absorbance and A_t_—absorbance at time t.

(5) $$D_{E}\left(\%\right)=\frac{A_{0}-A_{t}}{A_{0}}\times 100$$

### 4.3. Kinetic Studies

A pseudo-first-order reaction model was used to describe the decolorization of methylene blue. The kinetics of most organic substance degradation are most often described by a pseudo-first-order reaction model [52,53,54]. The pseudo-first-order model is expressed by Equation (6).

(6) −lnCtC0=kt

where C_t_—MB concentration at time t [ppm]; C_0_—initial MB concentration [ppm]; k—pseudo-first-order rate constant [1/min].

### 4.4. Radical Scavenger Tests

A free radical scavenging assay was used to evaluate the main oxidizing agents. This type of scavenger test can provide qualitative evidence. Nevertheless, additional mechanistic tools, such as electron paramagnetic resonance (EPR) spin-trapping analysis, competitive kinetic experiments, and identification of transformation products, would be required to determine the relative contribution of individual reactive species more precisely. Hydroquinone (HQ), tert-butyl alcohol (t-BuOH), and methanol (Me-OH) were used as radical scavengers of $O_{2}^{•-}$; •OH; both •OH and ${SO}_{4}^{•-}$, respectively. The dose of radical scavengers was set to 100 µM [55]. Radical scavengers were added at the beginning of the treatment process.

### 4.5. Statistical Analysis

An additional optimization test included a parametric analysis to examine the effects of individual parameters on the methylene blue decolorization rate during the advanced oxidation process in the presence of glucose and visible light. Analyzing the influence of the individual parameters on MB decolorization requires a high number of experimental runs and a description of the interaction effects of all the operational factors involved in the decolorization process.

To evaluate variable interactions and identify the best operational conditions for the decolorization effect (response), a design where every parameter level is combined with all other levels was employed (pH, glucose dose, SPS concentration, and initial dye concentration). For experimental optimization, a 3^4^ full factorial design consisting of 81 experimental runs was executed. Because each factor is tested at three levels, this design provides enough data points to fit a second-order (quadratic) polynomial model using response surface methodology (RSM). The complete set of independent variables along with their corresponding actual and coded levels is detailed in Table 3. The comprehensive 81-run matrix provided sufficient degrees of freedom to reliably estimate the model coefficients and map the response surfaces. The optimization was performed based on publications [56,57]. Statistica ver. 13 (TIBCO Software Inc., Palo Alto, CA, USA) was used for this purpose.

## 5. Conclusions

This study investigated the advanced decolorization of methylene blue by the SPS/Vis/glucose process. The response surface methodology confirmed that process efficiency is affected by pH, glucose dose, sodium persulfate concentration, and initial dye concentration. Selecting the appropriate doses of SPS and glucose, along with pH, enables the optimal balance of the process. Applying excess doses of SPS can result in adverse reactions between the sulfate anion and the hydroxyl radicals. On the other hand, exceeding the optimal glucose dose leads to lower decolorization levels due to the scavenging of oxidizing radicals. The highest decolorization level was obtained at the following parameters: SPS dose = 30 mM, glucose dose = 230 mM, pH = 4. The kinetics of the process followed pseudo-first-order (R^2^ = 97–99%). The radical scavenger test showed that both ${SO}_{4}^{•-}$ and •OH radicals are involved in methylene blue decolorization. This study presents an advancement in visible light-driven AOPs with particular emphasis on acidic conditions (pH = 4) representative of wool, alpaca, and mohair dyeing. Acidic conditions were beneficial, though another advantage of the process is its similar efficiency over a broad range of MB concentrations, from 1 to 10 ppm. Low energy consumption, thanks to the use of a low-power lamp (10 W), is another benefit. The technology is also metal-free, and it uses biodegradable organic promoters (glucose), as well as renewable sources of energy (visible light). Therefore, an AOP based on sodium persulfate, glucose, and visible light can be effectively applied for the pre-treatment of industrial wastewater containing dyes, particularly methylene blue. Although the study demonstrated the effectiveness of the process in decolorizing methylene blue, future practical studies require further evaluation of pollutant loads (COD, TOC), glucose and sulfate residues, and the generation of oxidation by-products.

## Figures and Tables

**Table 1:** Pseudo-first-order rate constants.

	First Stage (t = 0 ÷ 15 min)		Second Stage (t = 20 ÷ 70 min)	
	k_1_, 1/min	R^2^	k_2_, 1/min	R^2^
Control	0.0283	0.97	0.0101	0.99
T-BuOH	0.0202	0.97	0.0062	0.99
HQ	0.0247	0.97	0.0083	0.99
Me-OH	0.0132	0.98	0.0066	0.98

**Table 2:** Comparison with other advanced oxidation processes of methylene blue.

Process	Experimental Conditions	Efficiency [%]	Reference
Ozonation	pH = 4 C_0[MB]_ = 22 ppm Time = 60 min	61	[45]
Photocatalysis in the presence of magnetic TiO_2_/NiFe_2_O_4_/reduced graphene oxide nanocomposite	pH = 11 C_0[MB]_ = 10 ppm Time = 90 min Lamp = 500 W	71	[46]
UV/H_2_O_2_	pH = 4 C_0[MB]_ = 30 ppm Time = 30 min Lamp = 300 W	50	[47]
UV/H_2_O_2_	pH = 11 C_0[MB]_ = 50 ppm Time = 90 min Lamp = 11 W	50	[48]
Photo-Fenton	C_0[MB]_ = 20 ppm Time = 360 min Lamp = 10 W	72	[49]
Photocatalysis in the presence of magnetic zinc oxide/graphene/iron oxide-based catalyst	pH = 7 C_0[MB]_ = 10 ppm Time = 60 min Lamp = 70 W	54	[50]
Photocatalysis in the presence of CaWO_4_ under UV light	pH = 3 C_0[MB]_ = 100 ppm Time = 60 min Lamp = 80 W	28	[51]
SPS/Vis/glucose	pH = 4 C_0[MB]_ = 5 ppm Time = 70 min Lamp = 10 W	63	This study

**Table 3:** Experimental factors and their corresponding actual levels.

Factor	Unit	Factor Level		
		Low	Center	High
pH	-	4	6	9
Glucose dose	mM	200	230	260
SPS concentration	mM	25	30	35
Initial dye concentration	ppm	1	5	10

## Data Availability

The original contributions presented in this study are included in the article. Further inquiries can be directed to the corresponding author.

## Supplementary Materials

The following supporting information can be downloaded at: https://www.mdpi.com/article/10.3390/ijms27156620/s1.
