# Supplementary material for: Visible Light-Activated Persulfates Towards Dyes Decolorization: Performance, Kinetics, and Statistical Optimization
Source: Int J Mol Sci. 2026 Jul 24;27(15):6620. doi: 10.3390/ijms27156620 (PMC13466129; doi:10.3390/ijms27156620)
Supplement: Supplementary file 1 [file ijms-27-06620-s001.zip › ijms-4385146-supplementary.pdf]

Supplementary Materials

Visible Light-Activated Persulfates Towards Dyes Decolorization: Performance, Kinetics, and Statistical Optimization

Piotr Zawadzki * and Łukasz Pierzchała

Central Mining Institute—National Research Institute, Plac Gwarków 1, 40-166 Katowice, Poland; lpierzchala@gig.eu
* Correspondence: pzawadzki@gig.eu

Supplementary Materials Contains:

Number of pages: 3

Number of tables: 1

Table Captions:

Table S1. – Preliminary analysis and control procedures.

As part of the preliminary studies, basic control experiments were conducted, and the effects of various methylene blue (MB) decolorization configurations were examined: MB alone, visible light alone, SPS alone, glucose alone, SPS + visible light, SPS + glucose in the dark, glucose + visible light without SPS, and full SPS/Vis/glucose.

The results are summarized in Table S1.

The following conditions were used in the preliminary studies: $C_{0[MB]}$ = 5 ppm; pH = 6.0; Time = 70 min; SPS = 30 mM; Glucose = 230 mM.

The first stage of the study was to verify the individual effects of visible light, SPS, and glucose on the decolorization of methylene blue. The objective of this stage was to determine whether the presence of visible light, SPS, or glucose alone causes the dye to decolorize.

As shown in Table S1, the decolorization of MB in the presence of only visible light or only glucose does not cause the dye to decolorize. The combination "MB + glucose + visible light without SPS" was also tested, and the results confirmed that even the presence of two stimulants without SPS does not cause MB to decolorize. Conversely, the presence of SPS alone does not result in a significant decolorization effect (less than 10%). The possibility of MB decolorization without SPS activation is not a new phenomenon; however, it has significantly weaker oxidizing potential compared to activated SPS. The same was true for the "SPS + Visible light" system, where the decolorization effect was also negligible (less than 10%), since decolorization is possible without SPS activation, but the presence of visible light alone, without an activator, does not yield satisfactory results. A slightly higher decolorization effect was observed in the "MB + SPS + glucose in the dark" system (just under 16%) due to electron transfer from glucose to SPS, which acts as a stimulant for this oxidizing agent. However, without the presence of light (an additional energy source for SPS activation), the decolorization effect was unsatisfactory. Under experimental conditions, the combined SPS/Vis/glucose system exhibited more than twice the efficiency (32% decolorization effect), and this system was subsequently optimized.

Table S1. Preliminary analysis and control procedures.

Process	Experimental Conditions	Decolorization Effect [%]
MB alone	pH = 6.0<br>$C_{0[MB]}$ = 5 ppm<br>Time = 70 min	0.00±0.00
MB + visible-light alone	pH = 6.0<br>$C_{0[MB]}$ = 5 ppm<br>Time = 70 min	0.00±0.00
MB + SPS alone	pH = 6.0<br>$C_{0[MB]}$ = 5 ppm<br>Time = 70 min<br>SPS = 30 mM	9.87±1.04
MB + glucose alone	pH = 6.0<br>$C_{0[MB]}$ = 5 ppm<br>Time = 70 min<br>Glucose = 230 mM	0.00±0.00
MB + SPS + visible light	pH = 6.0<br>$C_{0[MB]}$ = 5 ppm<br>Time = 70 min<br>SPS = 30 mM	9.89±1.02
MB + SPS + glucose in the dark	pH = 6.0<br>$C_{0[MB]}$ = 5 ppm<br>Time = 70 min<br>SPS = 30 mM<br>Glucose = 230 mM	15.61±1.78
MB + glucose + visible light without SPS	pH = 6.0<br>$C_{0[MB]}$ = 5 ppm<br>Time = 70 min<br>Glucose = 230 mM	0.00±0.00
SPS/Vis/glucose	pH = 6.0<br>$C_{0[MB]}$ = 5 ppm<br>Time = 70 min<br>SPS = 30 mM<br>Glucose = 230 mM	32.00±2.21
